# Association of visual hallucinations with very mild degenerative dementia due to dementia with Lewy bodies

**DOI:** 10.1371/journal.pone.0205909

**Published:** 2018-10-15

**Authors:** Wei Lin, Yuan-Chang Xie, Po-Ya Cheng, Ling-Ying Dong, Guang-Uei Hung, Pai-Yi Chiu

**Affiliations:** 1 Department of Neurology, Chang Bin Show Chwan Memorial Hospital, Changhua, Taiwan; 2 Department of Neurology, Show Chwan Memorial Hospital, Changhua, Taiwan; 3 Department of Nuclear Medicine, Chang Bing Show Chwan Memorial Hospital, Changhua, Taiwan; Oslo Universitetssykehus, NORWAY

## Abstract

**Background and purpose:**

Complex, well-formed, and detailed visual hallucinations (VHs) are among the core clinical features of dementia with Lewy bodies (DLB). We investigated the diagnostic value of VHs in different types of very mild degenerative dementia.

**Methods:**

Participants were required to complete a structured interview form recording their basic data, clinical history, neuropsychological tests, and neuropsychiatric symptoms. Basic demographic characteristics of the participants were summarized and compared. The frequency and association factors of VHs were compared among three major degenerative dementia groups, namely, Alzheimer’s disease (AD), Parkinson’s disease dementia (PDD), and DLB.

**Results:**

A total of 197 patients with dementia and a clinical dementia rating of 0.5 were investigated, comprising 124 with AD, 35 with PDD, and 38 with DLB. A significantly higher frequency of VHs was found in the DLB group compared with the other groups (DLB, PDD, and AD = 31.6%, 11.4%, and 4.0%; *p* < 0.001). A multivariable logistic regression test for associations of positive VHs revealed that DLB was the only independently predictive factor (odds ratio: 13.62; *p* < 0.001).

**Conclusion:**

Our findings revealed a high diagnostic value of VHs in very mild degenerative dementia. VHs in this stage of dementia were significantly associated with DLB, and more than 30% of patients with very mild dementia caused by DLB presented with VHs.

## Introduction

Dementias associated with Lewy bodies are traditionally classified as dementia with Lewy bodies (DLB) or Parkinson’s disease dementia (PDD). The clinical characteristics of PDD and DLB are similar, mainly because of their shared neuropathology. These two diseases are also classified as Lewy body dementia (LBD) because Lewy-related pathology (intraneuronal aggregates of misfolded a-synuclein) is a hallmark of both diseases [1–3].

Neuropsychiatric symptoms (NPSs) such as delusions and hallucinations are commonly present in different types of dementia, including Alzheimer’s disease (AD) and LBD [4–7]. However, manifestations of NPSs vary among dementia types. For example, delusions occur early and for almost the entire course of all dementia types [6], whereas visual hallucinations (VHs) vary among dementia types with regard to their frequency, association with dementia severity, and contents [4–7]. One study reported that VHs occurred with high frequency and in earlier stages of DLB, but not in AD [3]. VHs are present in at least 60% of patients with DLB [8, 9], whereas their comorbidity with PDD is slightly lower, with similar contents [4–7, 10]. Typically, patients with LBD perceive complex, well-formed images of people, animals, or objects, which may recur several times a day. Studies of VHs in patients with DLB or PDD have revealed that, unlike for those with AD, VHs could occur prior to dementia onset, that is, in the mild cognitive impairment (MCI) stage [11].

Psychotic symptoms are also risk factors for the evolution of PD to PDD. However, studies on the frequency of VHs in PD-MCI or mild PDD are few, and comparisons between PDD and DLB concerning VHs in the very early stage of dementia remain insufficient. Given how common VHs are in LBD, it would be useful to know if their occurrence early in the course of dementia can help to differentiate between DLB, PDD, and AD. To achieve this aim, we enrolled a relatively large sample of patients with DLB and very mild (0.5) dementia according to the clinical dementia rating (CDR) scale and compared them with patients with PDD and AD.

## Methods

### Participants

We conducted this retrospective study using a register-based database of all patients who visited our hospital’s dementia clinic from October 1, 2015 to September 30, 2017. The demographic data included age, disease onset age, sex, education, disease duration, and use of antipsychotics or antiparkinsonians at the time of visit. A dementia diagnosis was made according to the major neurocognitive disorder criteria in the fifth edition of the Diagnostic and Statistical Manual of Mental Disorders (DSM-V). Diagnosis of PDD was made according to the clinical criteria for probable PDD developed by the Movement Disorder Society (MDS) in 2007 [10]. Diagnosis of DLB was made according to the revised consensus criteria for probable DLB developed in the third report of the DLB consortium [3]. To avoid the contribution of VHs in DLB diagnosis, we excluded VHs from the core DLB diagnostic features in the consensus criteria; more specifically, only fluctuation and parkinsonism were regarded as core features for a DLB diagnosis. Diagnosis of AD was made according to the criteria for dementia due developed by the diagnosis of dementia due to Alzheimer’s disease: recommendations from the National Institute on Aging-Alzheimer’s Association workgroups on diagnostic guidelines for Alzheimer’s disease [11].

### Assessment of VHs and other NPSs

All patients and their main caregivers were interviewed by a trained neuropsychologist for assessment of the 12-item Neuropsychiatric Inventory (NPI), based on observations within the past month. The NPI is a validated, standardized, and widely used instrument developed specifically for neuropsychiatric symptoms of dementia [12, 13]. The 12-item NPI comprises delusions, hallucinations, agitation, depression, anxiety, euphoria, apathy, disinhibition, irritation, aberrant motor behavior, night behavior, and eating/appetite behavior. All the items were rated for symptom frequency from 1 (occasionally) to 4 (very frequently), for symptom severity from 1 (mild) to 3 (severe), and for caregiver burden from 0 (none) to 5 (extreme) [12].

### Assessment of disease severity and cognitive function

The global severity of dementia was assessed according to the CDR scale and CDR sum of boxes (CDR-SB) [14]. To differentiate from MCI, only patients with CDR scores of 0.5 with impaired daily function were enrolled for assessment. Impaired daily function was defined by a total score of <7 on the Instrumental Activities of Daily Living Scale [15]. Cognitive function was assessed using the Montreal Cognitive Assessment (MoCA) [16] and the Cognitive Abilities Screening Instrument (CASI) [17]. Cognitive tests for all patients were performed by three trained neuropsychologists. Dementia and its subtypes were agreed upon by consensus among two neurologists, one geriatric psychiatrist, one doctor of nuclear medicine, and one neuropsychologist. All patients received cerebral CT or MRI scans and blood screening tests for dementia workup. Some patients also received dopamine transporter imaging using a Tc-99m TRODAT-1 SPECT because of parkinsonian symptoms.

### Data analysis

The Chinese version of SPSS version 19.0 for Windows (IBM, SPSS Inc., Chicago, IL, USA) was used for statistical analyses. Comparisons among the PDD, DLB, and AD groups for demographic data, CDR-SB, CASI, MoCA, and the NPI composite score (frequency × severity) were analyzed using one-way analysis of variance together with either Bonferroni or Dunnett T3 post hoc analysis, according to the homogeneity of variance. Sex, clinical features, and the use of antipsychotics or antiparkinsonians were analyzed using a chi-squared test. Multiple logistic regression analysis was used to compare associations for all patients, and the following factors were also compared between the VH+ and VH− groups: diagnosis, disease duration, age, sex, education, and MoCA. A *p* value of less than 0.05 was considered statistically significant.

### Ethical considerations

The participants were selected from a register-based health care system database in Taiwan. The study design was retrospective, and the data were analyzed anonymously. The Committee for Medical Research Ethics of Show Chwan Memorial Hospital reviewed the study, and the Data Inspectorate approved it.

## Results

A total of 197 individuals were examined, including 124, 35, and 38 patients with AD, PDD, and DLB, respectively. Among all patients, 104 (52.8%) were female, and 93 (47.2%) were male. Table 1 presents the demographic data of the three dementia groups. Comparisons of clinical and neuropsychiatric data revealed that the PDD group had a longer disease duration (*f* = 36.93; *p* < 0.001) and a higher rate of antiparkinsonian drug use (*f* = 53.17; *p* < 0.001). The DLB group had a significantly higher frequency of VHs compared with other groups (DLB = 31.6%; PPD = 11.4%; AD = 4.0%; *f* = 23.20; *p* < 0.001). It also had a higher total NPI score (*f* = 15.56; *p* < 0.001) and a higher rate of REM sleep behavior disorder (RBD; *f* = 62.66; *p* < 0.001). Both the PDD and DLB groups had a higher rate of abnormal dopamine transporter uptake (DaT abN; *f* = 28.41; *p* < 0.001)

**Table 1:** Comparison of demographic data among the PDD, DLB, and AD groups.

	PDD	DLB	AD	*f*/*χ*^2^	*p*	Post hoc/ paired comparison
*N*	35	38	124			
Age, year (SD, range)	72.8 (8.4, 51–85)	74.1 (9.2, 49–92)	74.9 (8.6, 54–91)	0.84	NS	PDD = DLB = AD
Female, *N* (%)	16 (45.7)	16 (42.1)	72 (58.1)	3.83	NS	PDD = DLB = AD
Education, years (SD, range)	5.9 (4.3, 0–16)	5.1 (4.7, 0–16)	5.8 (3.6, 0–16)	0.48	NS	PDD = DLB = AD
Disease duration, years (SD, range)	7.1 (5.9, 1.0–30.0)	1.9 (1.8, 0.1–8.0)	2.1 (2.2, 0.1–15.0)	36.93	<0.001	PDD > DLB = AD
CDR-SB (SD, range)	2.9 (0.7, 1.5–4.5)	2.8 (1.0, 1.5–6.0)	2.6 (0.8, 1.5–4.5)	2.52	NS	PDD = DLB = AD
MoCA (SD, range)	15.6 (8.4, 6–28)	15.2 (5.7, 6–28)	15.8 (5.9, 4–28)	0.17	NS	PDD = DLB = AD
CASI (SD, range)	71.3 (11.8, 53–93)	73.8 (10.1, 51–93)	73.0 (11.6, 40–94)	0.45	NS	PDD = DLB = AD
NPI sum (SD, range)	3.9 (3.9, 0–14)	10.6 (9.1, 0–41)	4.3 (5.8, 0–29)	15.56	<0.001	DLB > PDD = AD
CHEIs, *N* (%)	1 (2.9)	3 (7.9)	17 (13.7)	3.75	NS	PDD = DLB = AD
Antipsychotics, *N* (%)	5 (14.3)	3 (7.9)	11 (8.9)	1.08	NS	PDD = AD = DLB
Antiparkinsonians, *N* (%)	16 (45.7)	7 (18.4)	1 (0.8)	53.17	<0.001	PDD > DLB > AD
VH, *N* (%)	4 (11.4)	12 (31.6)	5 (4.0)	23.20	<0.001	DLB > PDD = AD
Fluctuation, *N* (%)	1 (2.9)	12 (31.6)	8 (6.5)	22.00	<0.001	DLB > PDD = AD
Parkinsonism, *N* (%)	35 (100)	32 (84.2)	9 (7.3)	140.45	<0.001	PDD = DLB > AD
RBD, *N* (%)	7 (20.0)	22 (57.9)	4 (3.2)	62.66	<0.001	DLB > PDD > AD
DaT abN, *N* (%)*	21 (100)	13 (81.3)	0 (0.0)	28.41	<0.001	PDD = DLB > AD

PDD: Parkinson’s disease dementia; DLB: dementia with Lewy bodies; AD: Alzheimer’s disease; NS: nonsignificant.

CDR-SB: sum of boxes on the clinical dementia rating Scale; CASI: Cognitive Abilities Screening Instrument; MoCA: Montreal Cognitive Assessment; NPI: Neuropsychiatric Inventory; CHEIs: currently using cholinesterase inhibitors (CHEIs); Antipsychotics: currently using antipsychotics; Antiparkinsonians: currently using antiparkinsonians; VH: visual hallucinations; RBD: REM sleep behavior disorder; DaT abN: abnormal uptake in dopamine transporter imaging.

*Number of patients receiving DaT in the PDD, DLB, and AD groups was 21, 16, and 5, respectively.

**Fig 1:**
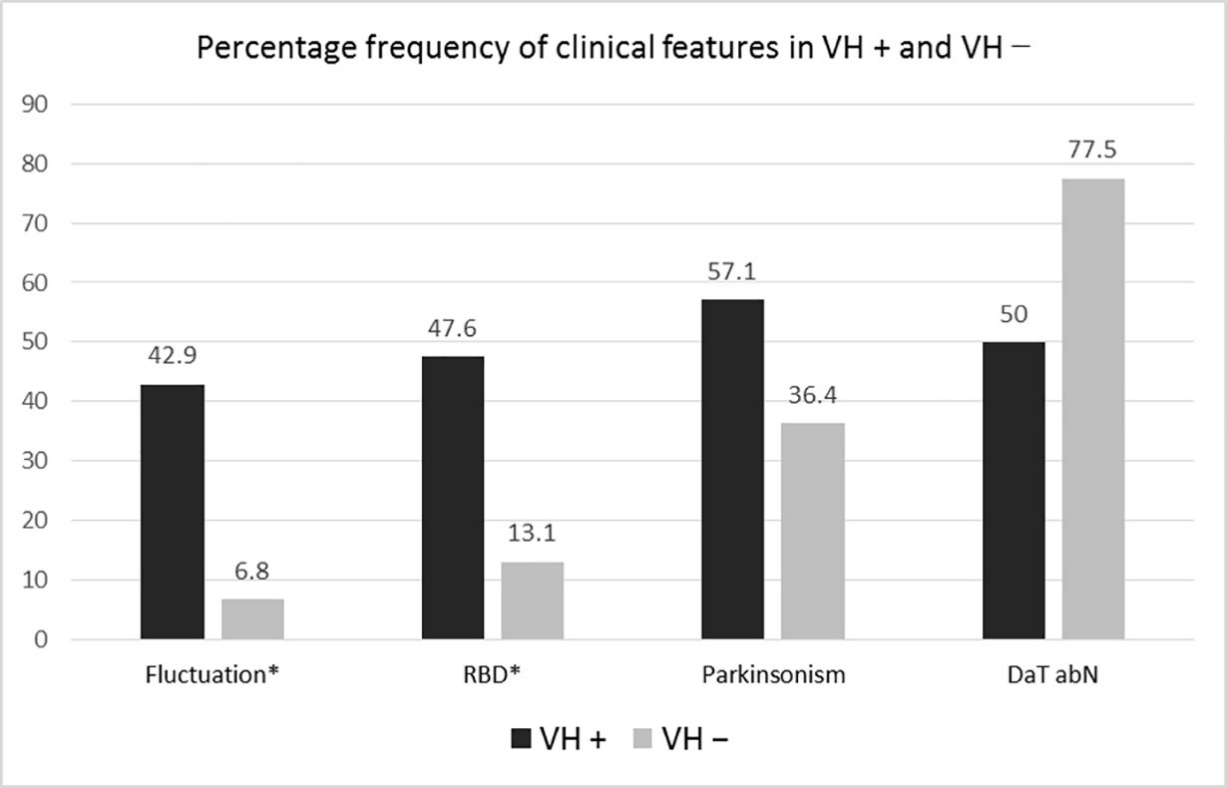
Comparison of frequency of clinical features between VH+ and VH− patients. VH: visual hallucinations; RBD: REM sleep behavior disorder; DaT abN: abnormal dopamine transporter uptake. * *p* < 0.05.

Fig 1 illustrates different associations of major clinical features for DLB diagnosis between the VH+ and VH− groups. The VH+ group had a significantly higher rate of cognition fluctuation (*χ*2 = 25.59; *p* < 0.001) and RBD (*χ*^2^ = 16.06; *p* < 0.001).

Table 2 presents the results of a multivariable logistic regression test for the association of positive VHs, revealing that a DLB diagnosis was the only independent predictor of VHs (odds ratio (OR): 13.62; *p* < 0.001).

**Table 2:** Multivariate risk estimates (ORs) for the VH+ group (*n* = 21, 10.7%) compared with the VH− group (*n* = 178, 89.3%).

Characteristics		No (%)	OR (95% CI)	*p*
Diagnosis	DLB	38 (19.3)	13.62 (3.50–53.15)	<0.001
	PDD	35 (17.8)	2.93 (0.60–14.30)	NS
	AD	124 (62.9)	1	
Disease duration, years	≤3	116 (58.9)	0.79 (0.23–2.69)	NS
	>3	81 (41.1)	1	
Sex	Female	104 (52.8)	2.91 (0.87–9.71)	NS
	Male	93 (47.2)	1	
Age, year s	≤75	100 (50.8)	0.55 (0.10–1.24)	NS
	>75	97 (49.2)	1	
Education, years	≤5	71 (36.0)	0.81 (0.59–1.11)	NS
	>5	126 (64.0)	1	
NPI	≤4	119 (60.4)	0.47 (0.16–1.40)	NS
	>5	78 (39.6)	1	
MoCA	≤15	95 (48.2)	1.47 (0.40–5.40)	NS
	>15	102 (51.8)	1	

OR: Odds ratio; VH: visual hallucinations; CI: confidence interval; NS: nonsignificant; DLB: dementia with Lewy bodies; PDD: Parkinson’s disease dementia; AD: Alzheimer’s disease; NPI: Neuropsychiatric Inventory; MoCA: Montreal Cognitive Assessment.

**Fig 2:**
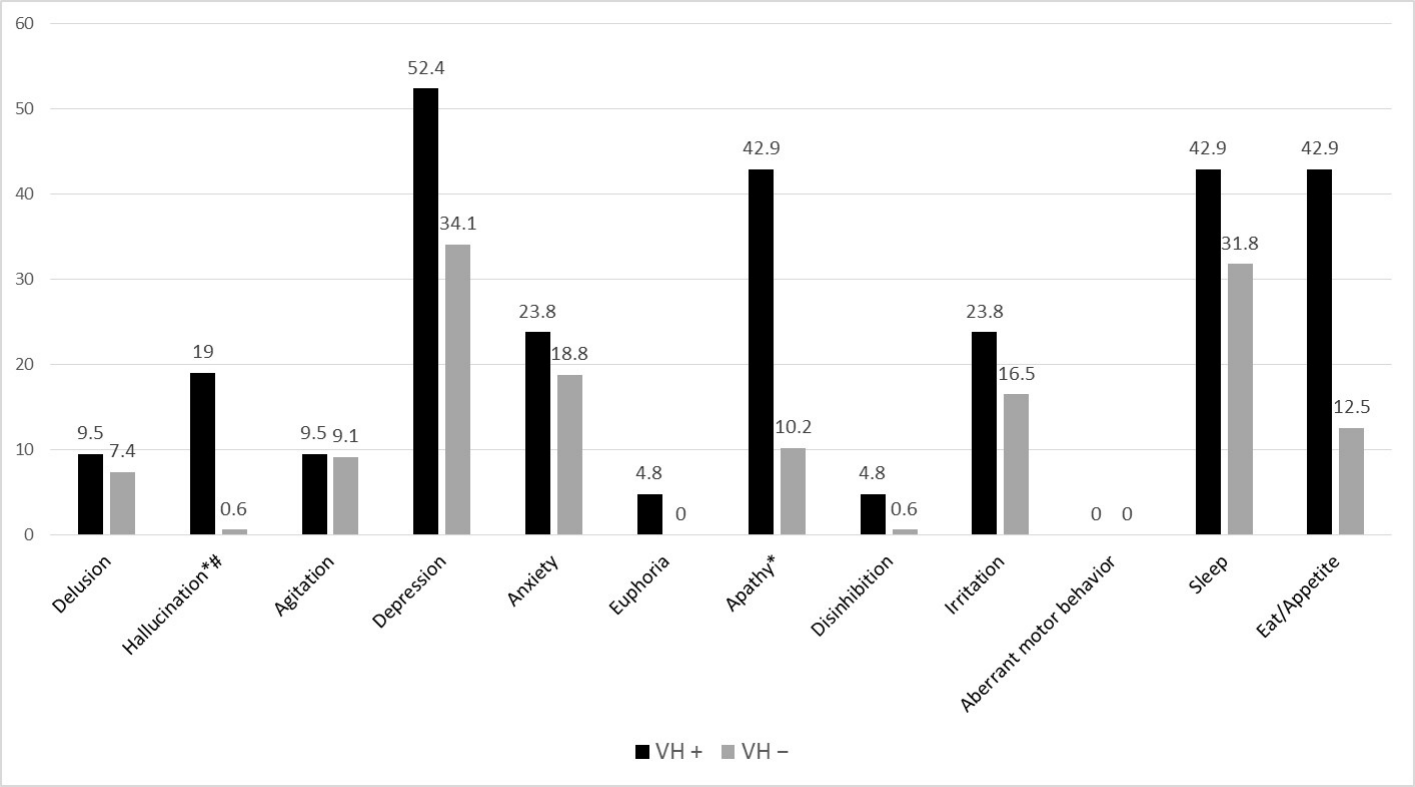
Comparison of frequency of each NPI domain between VH+ and VH− patients. # Hallucination in nonvisual domain. * *p* < 0.001.

The presence of other types of hallucinations, namely, auditory (19% versus 2.3%) and crawl (4.8% versus 0%), was significantly higher in the VH+ group than in the VH− group. No nonvisual hallucinations were found in either other group. One or more psychiatric symptoms were reported in 85.7% of the VH+ group and 69.3% of the VH− group. Fig 2 presents a comparison of the 12 NPI items between the two groups. The VH+ group had a significantly higher occurrence of other hallucinations (*χ*^2^ = 25.90; *p* < 0.001) and apathy (*χ*^2^ = 16.89; *p* < 0.001).

## Discussion

Relatively few studies have directly compared the manifestations of neuropsychiatric symptoms of DLB with those of other dementia types in the early disease stage [18, 19]. In this study, the AD, PDD, and DLB groups were well matched in terms of age and degree of cognitive impairment at baseline. One major finding of this study was that patients with DLB had significantly higher NPI scores and higher rates of VHs and RBD. These results highlight how common visuoperceptual disturbances are in early DLB compared with other degenerative dementias. Moreover, our findings are useful for distinguishing DLB from AD and PDD in extremely early disease stages.

We also found that patients with very mild dementia caused by DLB had significantly higher total NPI scores than those with PDD and AD, despite all three groups of patients having similar cognitive and baseline CDR scores. This suggests that caregivers of patients with DLB had higher distress levels from the more severe NPSs associated with DLB, a finding consistent with other published data [20, 21]. In a clinicopathological study, patients with MCI who subsequently developed DLB had more frequent delirium and VHs compared with patients who developed AD [22].

Comparison of the clinical presentation among patients with or without VHs in our study yielded other notable findings. In the NPI individual domain, the VH+ group had significantly higher scores for apathy (*p* < 0.001) than the VH− group. In addition, patients with DLB exhibited a significantly greater severity of delusions, hallucinations, anxiety, and sleep disturbances than those with AD and PDD. These findings were consistent with those of other studies [23, 24].

Peavy et al. [25] found that patients with LBD could have another common neuropsychological deficit of executive impairment and behavioral manifestations of frontal lobe dysfunction. They hypothesized that frontal-subcortical circuits associated with frontal lobe behaviors were affected early in AD but in later stages of DLB, which could aid in differentiating these two diseases. “Moderately demented patients with DLB were rated as exhibiting more apathy, behavioral executive dysfunction, and disinhibition than mildly demented DLB patients, whereas behavioral ratings did not diverge based on dementia severity in the AD.” [25]. This diverges from our study’s finding that apathy was prominent even in the very mild dementia stage, which was also consistent with the study by Donaghy et al. [26], who demonstrated much higher apathy and greater executive dysfunction in the predementia stage of DLB than in that of AD. Therefore, we disagree with the hypothesis that frontal-subcortical circuits associated with frontal lobe behaviors are not affected early in DLB.

Several methodological issues limit the interpretation of this study’s results. First, the diagnosis was made solely on a clinical basis without histopathological confirmation; therefore, some uncertainty necessarily exists about the degree of misclassification. In addition, we only used parkinsonism, fluctuations, and RBD as primary diagnostic features. Because this does not completely reflect how patients are diagnosed according to the McKeith criteria, it could be problematic and result in biased diagnoses of DLB. Second, the study design might have been suboptimal because behavioral disturbances can fluctuate and may not be present at every examination.

In conclusion, our findings revealed that VHs had a high diagnostic value in very mild degenerative dementia. VHs were highly associated with DLB in this stage of dementia, with more than 30% of patients with very mild dementia caused by DLB presenting with VHs. Further study is warranted to confirm the clinical utility of VHs in differentiating patients with very early degenerative dementia.

## References

[1] Cummings JL. Intellectual impairment in Parkinson's disease: clinical, pathologic, and biochemical correlates. Journal of geriatric psychiatry and neurology. 1988;1(1):24–36. Epub 1988/01/01. . 2908099 10.1177/089198878800100106

[2] Aarsland D, Andersen K, Larsen JP, Lolk A, Kragh-Sorensen P. Prevalence and characteristics of dementia in Parkinson disease: an 8-year prospective study. Archives of neurology. 2003;60(3):387–92. Epub 2003/03/14. . 12633150 10.1001/archneur.60.3.387

[3] McKeith IG, Dickson DW, Lowe J, Emre M, O'Brien JT, Feldman H, et al Diagnosis and management of dementia with Lewy bodies: third report of the DLB Consortium. Neurology. 2005;65(12):1863–72. Epub 2005/10/21. 10.1212/01.wnl.0000187889.17253.b1 . 16237129

[4] Johnson DK, Watts AS, Chapin BA, Anderson R, Burns JM. Neuropsychiatric profiles in dementia. Alzheimer disease and associated disorders. 2011;25(4):326–32. Epub 2011/11/17. 10.1097/WAD.0b013e31820d89b6 ; PubMed Central PMCID: PMCPMC3218373. 22086220 PMC3218373

[5] Lopez-Pousa S, Vilalta-Franch J, Garre-Olmo J, Pons S, Cucurella MG. [Characterisation and prevalence of the psychological and behavioural symptoms in patients with dementia]. Revista de neurologia. 2007;45(11):683–8. Epub 2007/12/01. . 18050101

[6] Hashimoto M, Yatabe Y, Ishikawa T, Fukuhara R, Kaneda K, Honda K, et al Relationship between Dementia Severity and Behavioral and Psychological Symptoms of Dementia in Dementia with Lewy Bodies and Alzheimer's Disease Patients. Dementia and geriatric cognitive disorders extra. 2015;5(2):244–52. Epub 2015/07/22. 10.1159/000381800 ; PubMed Central PMCID: PMCPMC4483492. 26195980 PMC4483492

[7] Sadak TI, Katon J, Beck C, Cochrane BB, Borson S. Key neuropsychiatric symptoms in common dementias: prevalence and implications for caregivers, clinicians, and health systems. Research in gerontological nursing. 2014;7(1):44–52. Epub 2013/10/02. 10.3928/19404921-20130918-01 ; PubMed Central PMCID: PMCPMC3909707. 24079749 PMC3909707

[8] Chiu PY, Teng PR, Wei CY, Wang CW, Tsai CT. Gender difference in the association and presentation of visual hallucinations in dementia with Lewy bodies: a cross-sectional study. International journal of geriatric psychiatry. 2018;33(1):193–9. Epub 2017/03/16. 10.1002/gps.4706 . 28295599

[9] McKeith IG, Boeve BF, Dickson DW, Halliday G, Taylor JP, Weintraub D, et al Diagnosis and management of dementia with Lewy bodies: Fourth consensus report of the DLB Consortium. Neurology. 2017;89(1):88–100. Epub 2017/06/09. 10.1212/WNL.0000000000004058 ; PubMed Central PMCID: PMCPMC5496518. 28592453 PMC5496518

[10] Emre M, Aarsland D, Brown R, Burn DJ, Duyckaerts C, Mizuno Y, et al Clinical diagnostic criteria for dementia associated with Parkinson's disease. Movement disorders: official journal of the Movement Disorder Society. 2007;22(12):1689–707; quiz 837. Epub 2007/06/02. 10.1002/mds.21507 . 17542011

[11] McKhann GM, Knopman DS, Chertkow H, Hyman BT, Jack CR Jr, Kawas CH, et al The diagnosis of dementia due to Alzheimer’s disease: recommendations from the National Institute on Aging-Alzheimer’s Association workgroups on diagnostic guidelines for Alzheimer’s disease. Alzheimers Dement 2011; 7: 263–69. 10.1016/j.jalz.2011.03.005 21514250 PMC3312024

[12] Cummings JL, Mega M, Gray K, Rosenberg-Thompson S, Carusi DA, Gornbein J. The Neuropsychiatric Inventory: comprehensive assessment of psychopathology in dementia. Neurology. 1994;44(12):2308–14. Epub 1994/12/01. . 7991117 10.1212/wnl.44.12.2308

[13] Assal F, Cummings JL. Neuropsychiatric symptoms in the dementias. Current opinion in neurology. 2002;15(4):445–50. Epub 2002/08/02. . 12151841 10.1097/00019052-200208000-00007

[14] Morris JC. The Clinical Dementia Rating (CDR): current version and scoring rules. Neurology. 1993;43(11):2412–4. Epub 1993/11/01. . 8232972 10.1212/wnl.43.11.2412-a

[15] Lawton MP, Brody EM. Assessment of older people: self-maintaining and instrumental activities of daily living. The Gerontologist. 1969;9(3):179–86. Epub 1969/01/01. . 5349366

[16] Nasreddine ZS, Phillips NA, Bedirian V, Charbonneau S, Whitehead V, Collin I, et al The Montreal Cognitive Assessment, MoCA: a brief screening tool for mild cognitive impairment. Journal of the American Geriatrics Society. 2005;53(4):695–9. Epub 2005/04/09. 10.1111/j.1532-5415.2005.53221.x . 15817019

[17] Teng EL, Hasegawa K, Homma A, Imai Y, Larson E, Graves A, et al The Cognitive Abilities Screening Instrument (CASI): a practical test for cross-cultural epidemiological studies of dementia. International psychogeriatrics. 1994;6(1):45–58; discussion 62. Epub 1994/01/01. . 8054493 10.1017/s1041610294001602

[18] Whitfield T, Sadiq D, Stevens T, Gonzales SC, Walker Z. Comparison of cognitive and clinical features between dlb-mci and ad-mci. Alzheimer's & Dementia: The Journal of the Alzheimer's Association. 2017;13(7):P1461 10.1016/j.jalz.2017.07.526

[19] Shin S, Lee JE, Hong JY, Sunwoo MK, Sohn YH, Lee PH. Neuroanatomical substrates of visual hallucinations in patients with non-demented Parkinson's disease. Journal of neurology, neurosurgery, and psychiatry. 2012;83(12):1155–61. Epub 2012/08/31. 10.1136/jnnp-2012-303391 . 22933812

[20] Bostrom F, Jonsson L, Minthon L, Londos E. Patients with Lewy body dementia use more resources than those with Alzheimer's disease. International journal of geriatric psychiatry. 2007;22(8):713–9. Epub 2006/12/30. 10.1002/gps.1738 . 17195278

[21] Ricci M, Guidoni SV, Sepe-Monti M, Bomboi G, Antonini G, Blundo C, et al Clinical findings, functional abilities and caregiver distress in the early stage of dementia with Lewy bodies (DLB) and Alzheimer's disease (AD). Archives of gerontology and geriatrics. 2009;49(2):e101–4. Epub 2008/12/17. 10.1016/j.archger.2008.10.001 . 19084284

[22] McKeith I, Mintzer J, Aarsland D, Burn D, Chiu H, Cohen-Mansfield J, et al Dementia with Lewy bodies. The Lancet Neurology. 2004;3(1):19–28. Epub 2003/12/25. . 14693108 10.1016/s1474-4422(03)00619-7

[23] Del Ser T, McKeith I, Anand R, Cicin-Sain A, Ferrara R, Spiegel R. Dementia with lewy bodies: findings from an international multicentre study. International journal of geriatric psychiatry. 2000;15(11):1034–45. Epub 2000/12/13. . 11113984 10.1002/1099-1166(200011)15:11<1034::aid-gps231>3.0.co;2-5

[24] Molano J, Boeve B, Ferman T, Smith G, Parisi J, Dickson D, et al Mild cognitive impairment associated with limbic and neocortical Lewy body disease: a clinicopathological study. Brain: a journal of neurology. 2010;133(Pt 2):540–56. Epub 2009/11/06. 10.1093/brain/awp280 ; PubMed Central PMCID: PMCPMC2822633. 19889717 PMC2822633

[25] Peavy GM, Salmon DP, Edland SD, Tam S, Hansen LA, Masliah E, et al Neuropsychiatric features of frontal lobe dysfunction in autopsy-confirmed patients with lewy bodies and "pure" Alzheimer disease. The American journal of geriatric psychiatry: official journal of the American Association for Geriatric Psychiatry. 2013;21(6):509–19. Epub 2013/04/10. 10.1016/j.jagp.2012.10.022 ; PubMed Central PMCID: PMCPMC3664517. 23567425 PMC3664517

[26] Donaghy PC, Taylor JP, O'Brien JT, Barnett N, Olsen K, Colloby SJ, et al Neuropsychiatric symptoms and cognitive profile in mild cognitive impairment with Lewy bodies. Psychologial Medicine. 2018;48(14):2384–90. 10.1017/S0033291717003956 29362011

